# Supplementary material for: Barley ROP-Interactive Partner-a organizes into RAC1- and MICROTUBULE-ASSOCIATED ROP-GTPASE ACTIVATING PROTEIN 1-dependent membrane domains
Source: BMC Plant Biol. 2020 Mar 2;20:94. doi: 10.1186/s12870-020-2299-4 (PMC7053138; doi:10.1186/s12870-020-2299-4)
Supplement: Supplementary file 1 — Additional file 1: Supplementary Figure S1. Barley RIPa interacts with itself in yeast. Bait- and prey-construct transformed yeast cells were dropped on either transformation-selected (SD -L-W) or interaction-selective (SD –L,-W,-A-H) medium. pGADT7 presents empty vector controls to exclude auto-activity of respective constructs. Supplementary Figure S2. Bimolecular fluorescence complementation of split YFP suggests direct interaction of RIPa and RAC1 in planta. A. In planta interaction of YFPN-RIPa and YPFC-RAC1 allows for YFP fluorescence complementation at the cell periphery and at MTs (column one). Fluorescence is faint when one of the split YFP proteins is expressed alone instead of being fused to RIPa or RAC1 (columns three and four). Fusion of YFPC to the dominant negative mutant RAC1-T28N does not interact with RIPa, and no fluorescence is observed (column two). Red fluorescing dsRED protein is localized in the cytoplasm and nucleoplasm and contrasts YFP fluorescence at the cell periphery. The pictures show projections of 20–30 optical sections through the epidermal cell at 2 μm increments. All signals have been recorded with the same microscope settings. B. Enlargement of the YFPN-RIPa –YFPC-RAC1 split YFP signal from column 1 in A. Brightness of the picture was enhanced by 40%. Supplementary Figure S3. Barley YFP-RIPa localizes to the cell periphery when co-expressed with CA RAC1 or CA RACB (left panels) and to speckles of unknown nature when co-expressed with DN RAC1 or DN RACB (right panels. Whole cell Z-stack images were taken 24 h after biolistic transformation of barley epidermal cells. The cytosolic marker mCherry was co-expressed to contrast the cytoplasm. Supplementary Figure S4. Interaction between MAGAP1 and RAC1. A. Barley MAGAP1 interacts with the barley type II ROP RAC1 in yeast. Bait- and prey construct-transformed yeast cells were dropped on either transformation-selected (SD -L-W) or interaction-selective (SD –L,-W,-A-H) medium. pGADT7 and [file 12870_2020_2299_MOESM1_ESM.pdf]

Figure S1

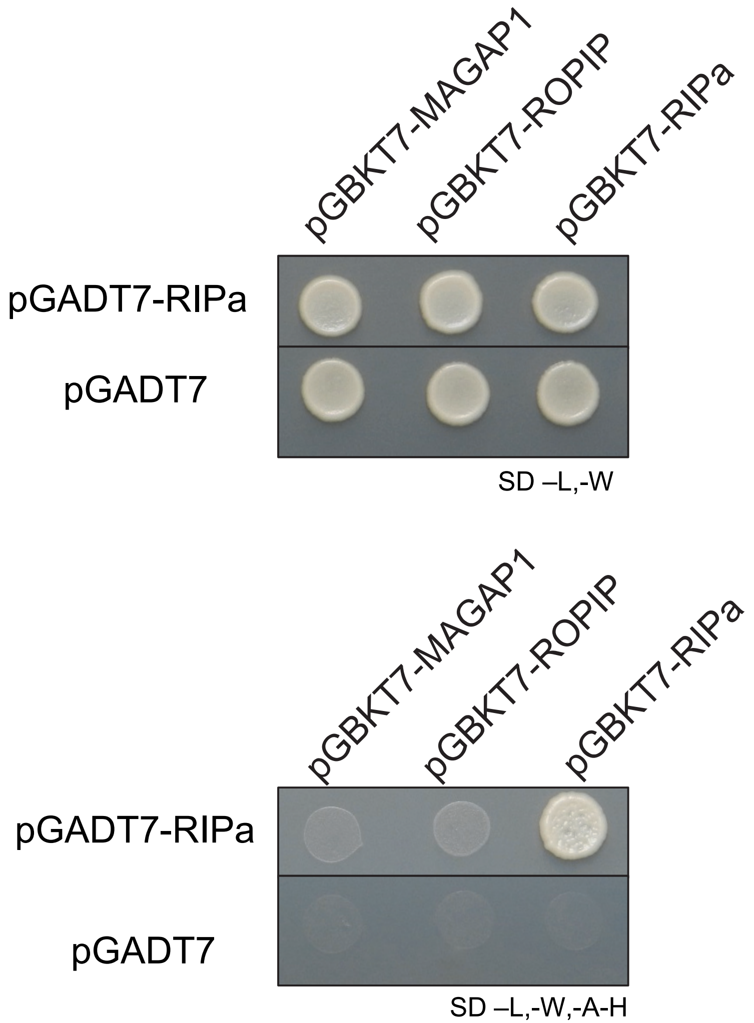

Supplementary Figure S1. Barley RIPa interacts with itself in yeast. Bait- and prey-construct transformed yeast cells were dropped on either transformation-selected (SD -L-W) or interaction-selective (SD –L,-W,-A-H) medium. pGADT7 presents empty vector controls to exclude auto-activity of respective constructs.

Figure S2

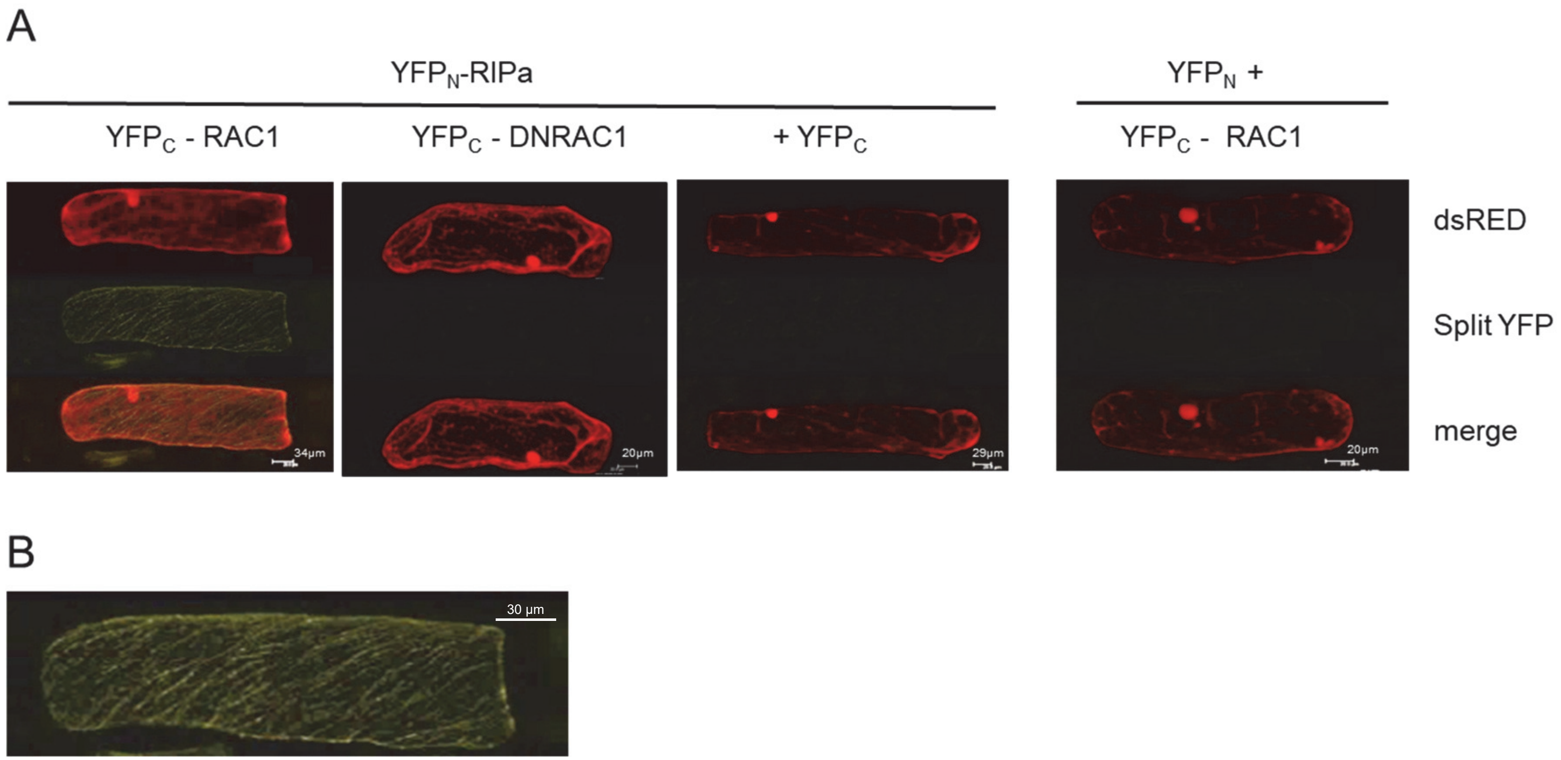

Supplementary Figure S2. Bimolecular fluorescence complementation of split YFP suggests direct interaction of RIPa and RAC1 in *planta*. A. *In planta* interaction of YFP$_N$-RIPa and YPF$_C$-RAC1 allows for YFP fluorescence complementation at the cell periphery and at MTs (column one). Fluorescence is faint when one of the split YFP proteins is expressed alone instead of being fused to RIPa or RAC1 (columns three and four). Fusion of YFP$_C$ to the dominant negative mutant RAC1-T28N does not interact with RIPa, and no fluorescence is observed (column two). Red fluorescing dsRED protein is localized in the cytoplasm and nucleoplasm and contrasts YFP fluorescence at the cell periphery. The pictures show projections of 20-30 optical sections through the epidermal cell at 2 µm increments. All signals have been recorded with the same microscope settings. B. Enlargement of the YFP$_N$-RIPa –YFP$_C$-RAC1 split YFP signal from column 1 in A. Brightness of the picture was enhanced by 40%.

Figure S3

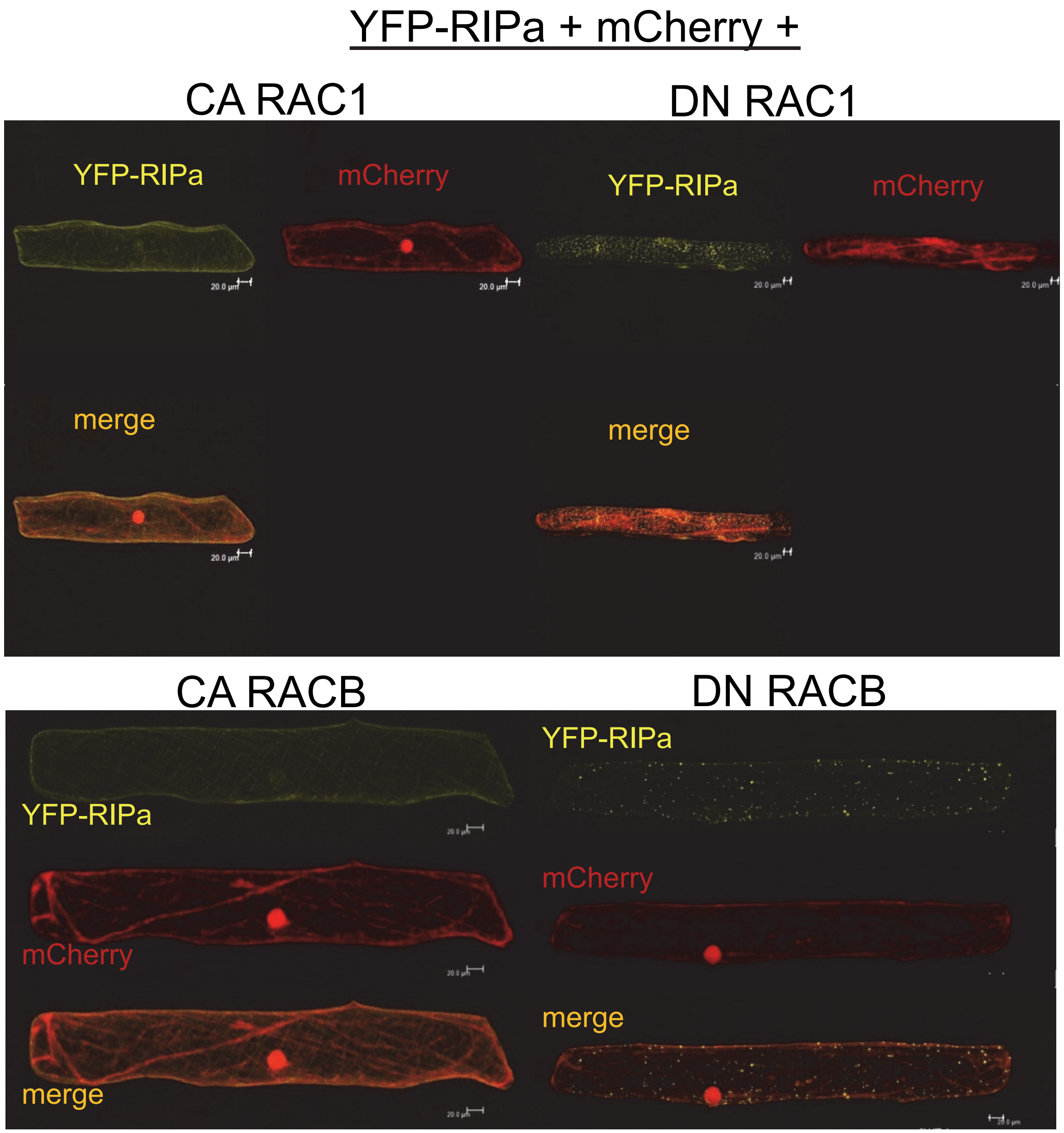

Supplementary Figure S3. Barley YFP-RIPa localizes to the cell periphery when co-expressed with CA RAC1 or CA RACB (left panels) and to speckles of unknown nature when co-expressed with DN RAC1 or DN RACB (right panels. Whole cell Z-stack images were taken 24 h after biolistic transformation of barley epidermal cells. The cytosolic marker mCherry was co-expressed to contrast the cytoplasm.

Figure S4

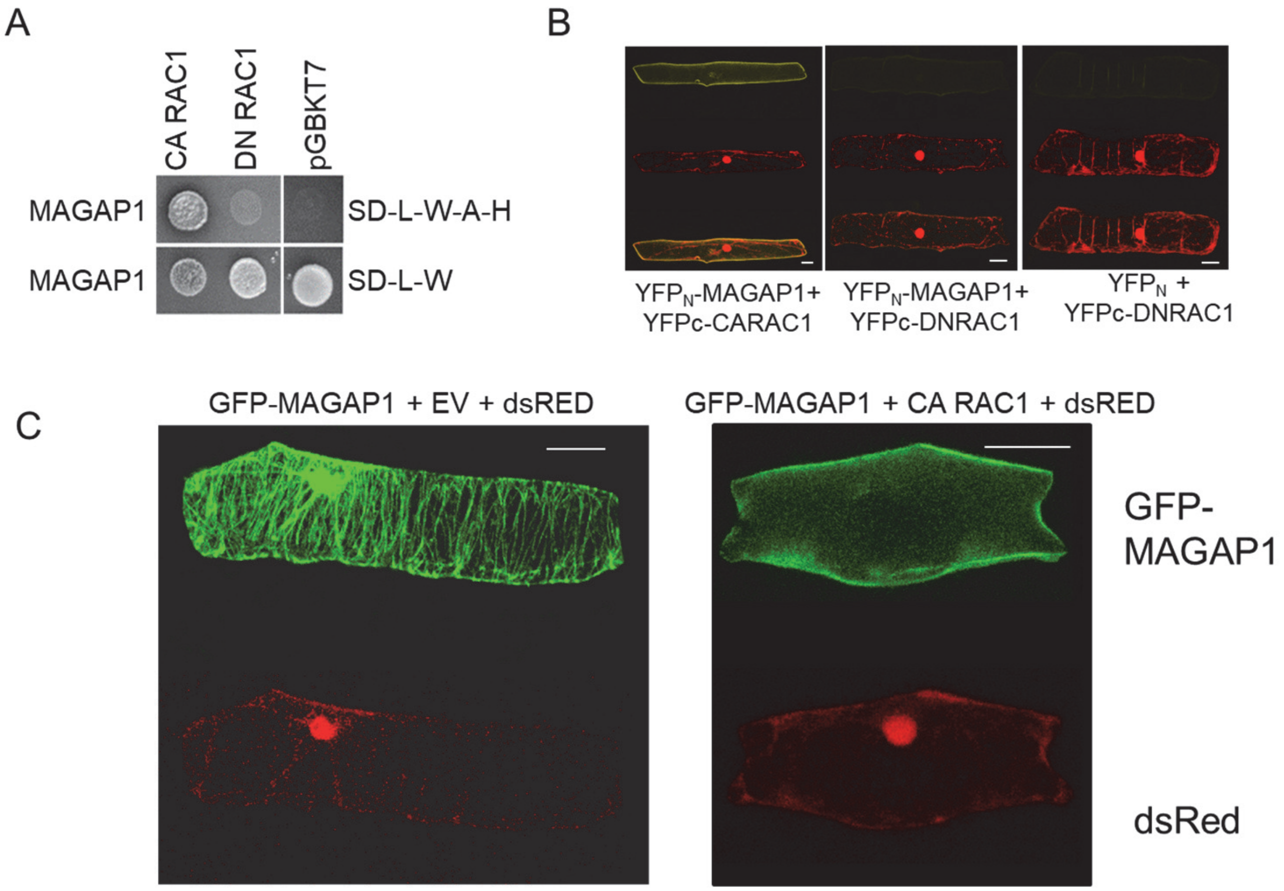

Supplementary Figure S4. Interaction between MAGAP1 and RAC1. A. Barley MAGAP1 interacts with the barley type II ROP RAC1 in yeast. Bait- and prey construct-transformed yeast cells were dropped on either transformation-selected (SD -L-W) or interaction-selective (SD –L,-W,-A-H) medium. pGADT7 and pGBKT7 represent an empty vector control to exclude auto-activity of the MAGAP1 construct. B. Bimolecular fluorescence complementation of split YFP suggests direct interaction of MAGAP1 and RAC1-GTP in *planta*. *In planta* interaction of YFP$_N$-MAGAP1 and YPF$_C$-CARAC1 allows for YFP fluorescence complementation at the cell periphery (column one). Fluorescence is faint when YPF$_C$-DNRAC1 is co-expressed instead activated RAC1 (columns two). In this case, background fluorescence complementation is similar to what is observed when free YFP$_N$ instead of YFP$_N$-MAGAP1 is coexpressed (column three). The pictures show projections of 20-30 optical sections through the epidermal cell at 2 µm increments. All signals have been recorded with the same microscope settings. C. Change of GFP-MAGAP1 localization upon co-expression of CA RAC1. Whole cell Z-stack images were taken 24 h after biolistic transformation of barley epidermal cells. GFP-MAGAP1 localizes to MTs but is recruited to the cell periphery upon co-expression of CA RAC1. EV, empty vector. Lower panel: The cytosolic marker DsRED was co-expressed. Bars represent 30 µm.

Figure S5

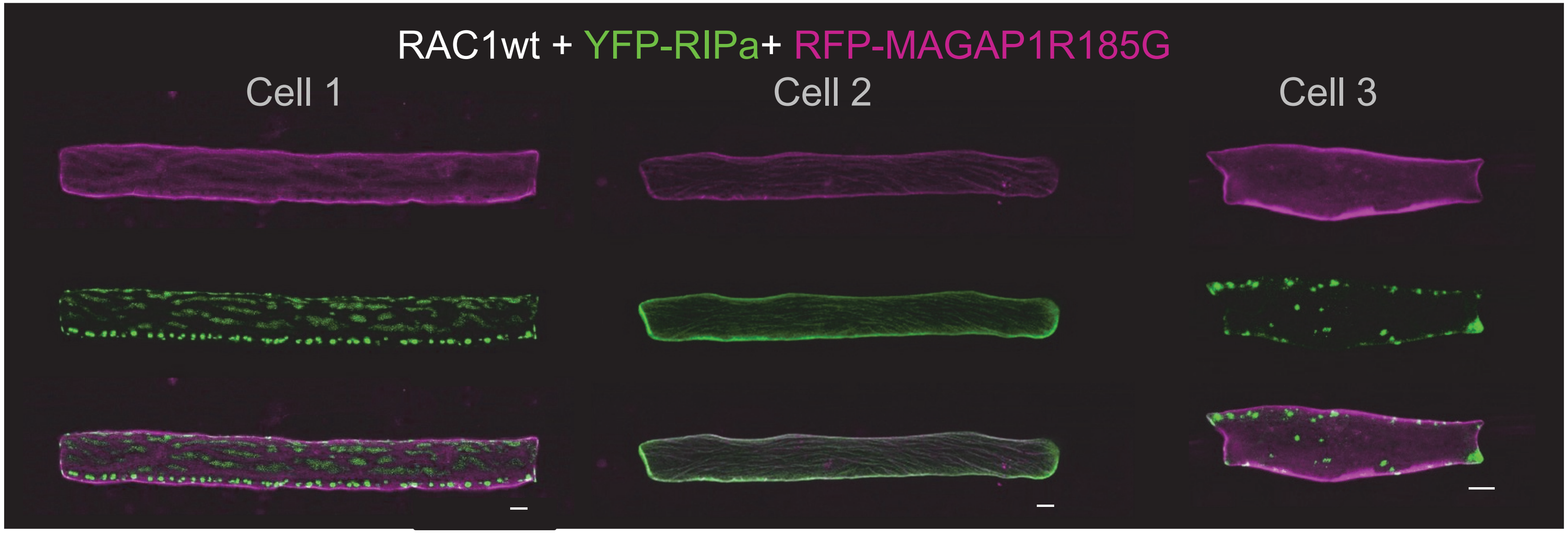

Supplementary Figure S5. Co-expression of RAC1 and the presumably GAP-inactive mutant MAGAP1R185G leads to inconsistent RIPa localization. Co-expression of fluorescent YFP-RIPa with untagged RAC1 and with RFP-MAGAP1R185G. Whole cell Z-stack images of three representative cells with diverse locasiation patterns of YFP-RIPa were taken 24 h after biolistic transformation of barley epidermal cells. Bars represent 20 µm.

Figure S6

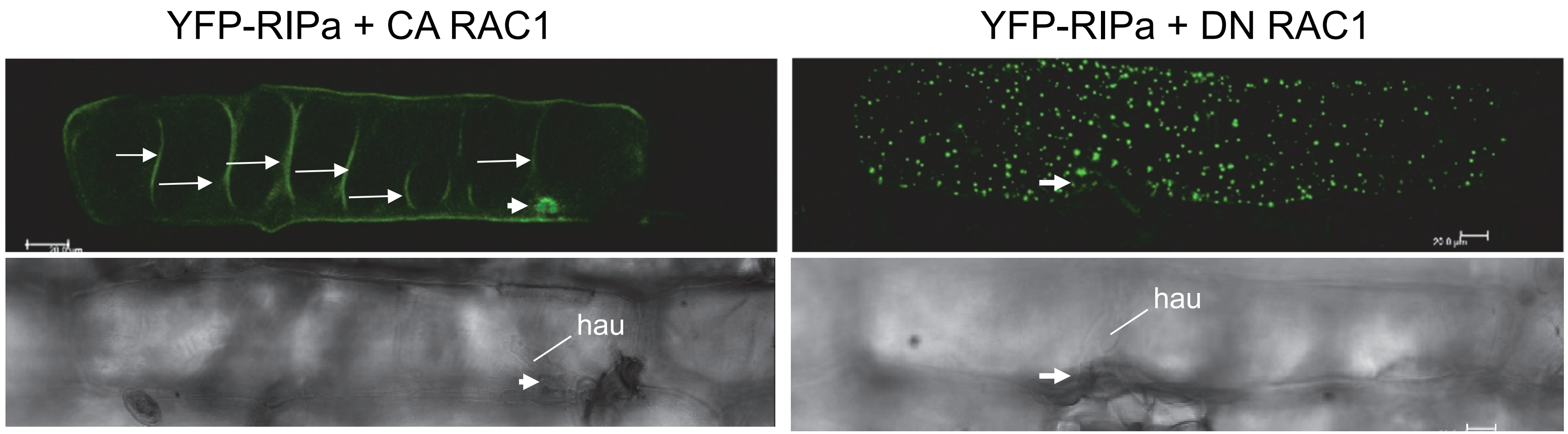

Supplementary Figure S6. YFP-RIPa localization at sites of fungal attack by *Bgh* but not when DN RAC1 is co-expressed. Whole cell Z-stack images were taken 28 h after biolistic transformation of barley epidermal cells and 23 h after inoculation. Additionally, untagged CA RAC1 or DN RAC1 are co-expressed. Brightness was enhanced by 20 % after imaging. Please note the fungal attack from an appressorium (app). Site of attack, arrow; hau; fungal haustorium. Long arrows mark plasma membrane folds at cell wall protrusions at the cell bottom facing mesophyll cells. Bars represent 20 µm.
